# The Kiss of Death: Serratia marcescens Antibacterial Activities against Staphylococcus aureus Requires Both *de novo* Prodigiosin Synthesis and Direct Contact

**DOI:** 10.1128/spectrum.00607-22

**Published:** 2022-04-18

**Authors:** Sungbin Lim, Jihun Bhak, Sungwon Jeon, Wonsik Mun, Jong Bhak, Seong Yeol Choi, Robert J. Mitchell

**Affiliations:** a Department of Biological Sciences, Ulsan National institute of Science and Technology (UNIST), Ulsan, South Korea; b Department of Biomedical Engineering, Ulsan National institute of Science and Technology (UNIST), Ulsan, South Korea; c Korean Genomics Center (KOGIC), Ulsan National Institute of Science and Technology (UNIST), Ulsan, South Korea; d Clinomics Inc., Ulsan, South Korea; e Personal Genomics Institute (PGI), Genome Research Foundation (GRF), Osong, South Korea; Ohio State University

**Keywords:** *Serratia marcescens*, *Staphylococcus aureus*, prodigiosin, membrane vesicles, type VI secretion system, *de novo* assembly, antibacterial, cell-to-cell contact

## Abstract

Prodigiosin possesses antibacterial activities, but as a highly hydrophobic compound, it raised the question about how Serratia marcescens introduce this compound to other microbes. Here, we demonstrate that the production of prodigiosin by newly isolated S. marcescens RH10 correlates with its antibacterial activity against a multidrug-resistant strain of S. aureus, with this pathogen’s viability decreasing 6-log over 24 h. While S. marcescens RH10 does secrete membrane vesicles that carry prodigiosin, this antibiotic was not active in this form, with 5 mg/L prodigiosin leading to only a 1.22-fold reduction in the S. aureus viability while the same quantity of purified prodigiosin led to a 2800-fold reduction. Contact assays, however, showed increased activity, with a 3-log loss in the S. aureus viabilities in only 6 h as long as *de novo* production of prodigiosin occurred. The role of prodigiosin was confirmed further by generating an isogenic Δ*pigA* mutant in S. marcescens RH10, based on the draft genome sequence reported here, to inhibit the synthesis of prodigiosin. In all experiments performed, this mutant was unable to kill S. aureus. Finally, the possibility that the type VI secretion system present in S. marcescens may also be important was also explored as it is known to be used by this strain to kill other microbes. The results here, however, found no obvious activity against S. aureus. In conclusion, the results presented here show prodigiosin requires both cell-to-cell contact and *de novo* synthesis for it to be effective as an antibiotic for its native host.

**IMPORTANCE** The antibacterial activities of prodigiosin are well-established but, as a hydrophobic molecule, the mechanisms used to introduce it to susceptible microbes has never been studied. We found here, in contrast to violacein, another hydrophobic antibiotic that can be transferred using membrane vesicles (MVs), prodigiosin is also carried from Serratia marcescens in MVs released but its resulting activities were severely mitigated compared to the freely added compound, suggesting it is more tightly bound to the MVs than violacein. This led us to hypothesize that cell-to-cell contact is needed, which we demonstrate here. As well, we show *de novo* synthesis of prodigiosin is needed for it to be effective. As violacein- and prodigiosin-producing bacterial strains are both beneficial to amphibians, where they help protect the skin against pathogens, the findings presented here provide an important ecological perspective as they show the mechanisms used differ according to the antibacterial produced.

## OBSERVATION

Prodigiosin is a vibrant red, tripyrrole pigment produced by different bacterial strains, including *Janthinobacterium* (1) and *Streptomyces* (2, 3), but is best characterized in Serratia marcescens (4), where the proteins responsible for its biosynthesis are encoded within the prodigiosin biosynthesis gene cluster (*pig*) (5). As a secondary metabolite, prodigiosin possesses a range of biological activities, as reviewed recently (6), but its antimicrobial activities are probably best known, with a number of bacterial pathogens reportedly being sensitive (6–9), including Bacillus cereus (10), Streptococcus pyogenes (11) and, in particular, Staphylococcus aureus (10, 11). While the bactericidal mechanisms of prodigiosin, including the production of reactive oxygen species (ROS) (10, 12), H^+^/CL^−^ transporter uncoupling (13) and membrane disruption attributed to prodigiosin’s strong hydrophobic character (14), have all been explored by several different groups, they do not explain how this compound is conveyed from its host to other microorganisms, and is active in natural environments (15–17).

**FIG 1:**
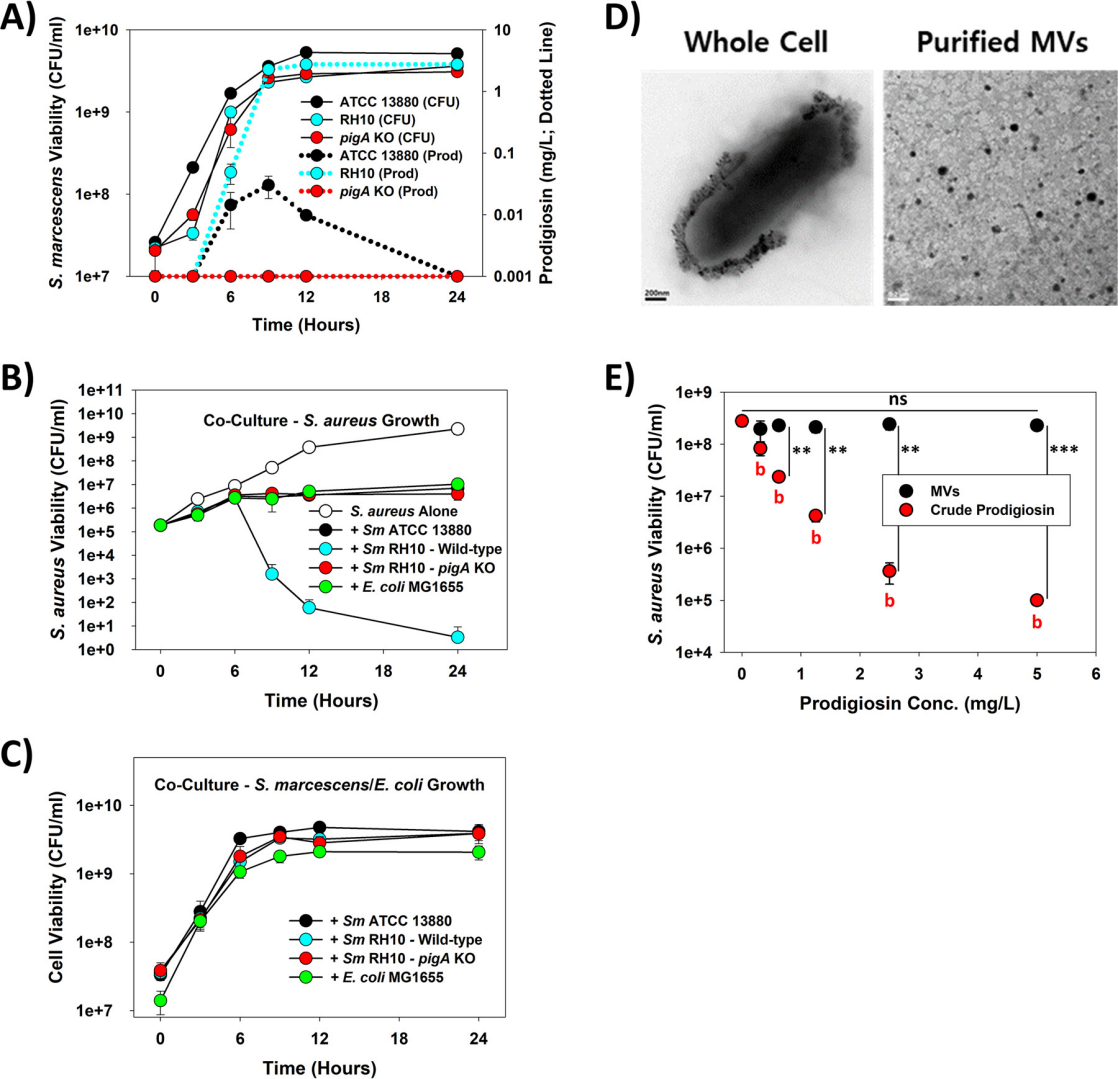
Killing of S. aureus by prodigiosin from S. marcescens is not mediated by membrane vesicles. (A) Growth (CFU/mL) and prodigiosin (Prod) production (mg/L) by different S. marcescens strains, showing considerably higher prodigiosin yields from S. marcescens RH10 compared with the type strain, i.e., S. marcescens ATCC 13880. (*n *= 3) (B) MDR S. aureus clinical isolate viabilities when grown in cocultures with the various S. marcescens strains or E. coli MG1655, showing only wild-type S. marcescens RH10 led to obvious killing of S. aureus after 6 h. (*n *= 3) (C) S. marcescens and E. coli MG1655 viabilities from the cocultures with the MDR S. aureus clinical isolate plotted in (B). (*n *= 3) (D) Transmission electron microscopic images of S. marcescens RH10 and its purified membrane vesicles. The size bars in both images are 0.2 μm. (E) Membrane vesicles do not effectively transport prodigiosin to S. aureus. Viability plot after a 6-h treatment showing freely added prodigiosin was significantly more bactericidal against S. aureus than when provided within the purified membrane vesicles. Significance between samples within the same group: ns, not significant; b (*P* < 0.01). Significance between groups: **, *P* < 0.01; ***, *P* < 0.001. (*n *= 3).

To explore this, we employed the newly isolated strain S. marcescens RH10 (Table S1; Accession Numbers SRR14952065, PRJNA741880) as it is much more proficient at synthesizing prodigiosin (Fig. S1 and S2) than the type strain, S. marcescens ATCC 13880 (Fig. 1A and S3). Characterization of both strains found prodigiosin productivities increased drastically as they entered the stationary phase, a result that is not surprising given the role quorum sensing plays in governing expression of the *pig* operon (5, 18). However, the maximum yields from S. marcescens RH10 (2.76 mg/L) were significantly greater, i.e., 91-fold higher (*P* = 0.00031), than those obtained with S. marcescens ATCC 13880 (0.03 mg/L) (Fig. 1A). This difference had significant effects on the resulting bactericidal activities of these two strains against a multidrug-/methicillin-resistant clinical strain of S. aureus (19).

As shown in Fig. 1B, the S. aureus viability decreased only when grown together with S. marcescens RH10, not S. marcescens ATCC 13880. In contrast, both S. marcescens strains grew well (Fig. 1C) and achieved cell densities that were similar than when grown alone (Fig. S4), showing the toxicity was unidirectional. More importantly, the drop in S. aureus viability was evident from 9 h (Fig. 1B), when S. marcescens RH10 began to produce mg levels of prodigiosin (Fig. 1A). To validate that this loss was due to prodigiosin and not some other secondary metabolite, an isogenic Δ*pigA* mutant of S. marcescens RH10 was constructed based on the draft genome sequence for this strain (Fig. S5–S8), rendering it pigmentless (Fig. 1A and S3). Performing the same growth tests with either this isogenic mutant or E. coli str. MG1655, which was selected as another prodigiosin-negative-control bacterial strain, we found neither had a negative impact on the S. aureus viability (Fig. 1B).

Although the data above established prodigiosin is responsible for the loss in S. aureus viability when grown together with S. marcescens RH10, this molecule is highly hydrophobic (Log_POW_ of 5.16) (14), obliging an explanation on how it is transferred to S. aureus. A previous study with violacein, a different secondary metabolite with antibiotic properties that is produced by other bacterial strains (i.e., *Chromobacterium* spp.) (19, 20) but not the S. marcescens strains employed here, can be transported from its host to S. aureus via membrane vesicles (MVs), where it kills this pathogen (21). Although S. marcescens RH10 likewise produced MVs (Fig. 1D) that also contained prodigiosin (Fig. S9), they possessed no detectable activities against S. aureus and were much less potent compared in tests with purified crude prodigiosin (i.e., a 1.22-fold versus 2800-fold reduction in S. aureus viability with 5 mg/L prodigiosin, respectively; Fig. 1E). This is further supported in Fig. S10, where filtered (0.22 μm) spent media from S. marcescens RH10 also had no overt antibacterial activity against S. aureus. However, the lower overall viabilities in these samples were lower compared against the tests performed with spent media from S. marcescens RH10 Δ*pigA*, suggesting the spent media from S. marcescens RH10 possessed some slight inhibitory activities. Related with this, we found that as S. marcescens RH10 grew, its supernatant did have some prodigiosin present, particularly at 9 h when it reached a maximum (Fig. S11), which may help explain this phenomenon. Taken together, however, these results demonstrate the majority of the prodigiosin fraction is not solubilized within the media or present in the MVs and, therefore, must still be associated with the bacterial cell and transferred via cell-to-cell contact.

**FIG 2:**
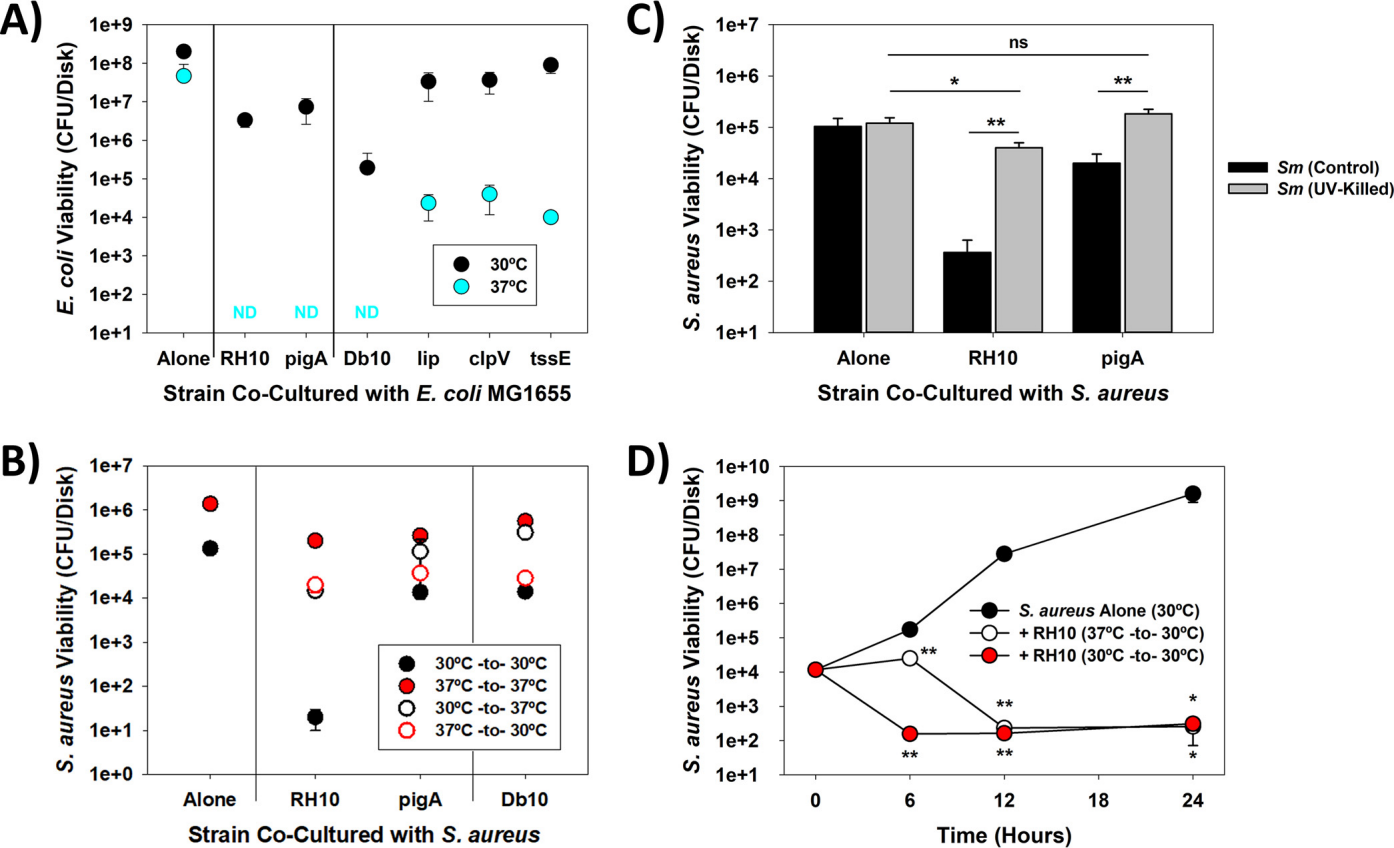
Prodigiosin’s bactericidal activities against S. aureus require direct cellular contact and *de novo* synthesis. (A) Type VI secretion system killing of E. coli MG1655 by S. marcescens strains RH10 and Db10 (and their isogenic mutants) at different temperatures. Note the higher bactericidal activities (stronger killing of E. coli MG1655) from all of the S. marcescens strains when cultured at 37°C. ND – Not Detected (<10 E. coli MG1655 CFU/disk). (*n *= 3) (B) The Type VI secretion system of S. marcescens is ineffective against S. aureus. Growth of S. marcescens initially at or performing this assay at 37°C reduced its activity against the MDR S. aureus isolate, in clear contrast to the results seen with E. coli MG1655 (A). This was due to inhibition or a delay in prodigiosin production, showing *de novo* synthesis of prodigiosin is necessary. Each of the viabilities were measured at 6 h. (*n *= 3) (C) UV-killing of S. marcescens RH10 and its isogenic Δ*pigA* mutant abolished much of their ability to kill S. aureus and inhibit its growth, respectively. These results further validate *de novo* synthesis of prodigiosin by S. marcescens is necessary for S. aureus killing. Ns, not significant; *, *P* < 0.05; **, *P* < 0.01. (*n *= 3) (D) Growth of S. marcescens RH10 at 37°C delays its bactericidal activities against S. aureus. Longer incubations of the direct contact cultures saw the prodigiosin quantities increase considerably after 6 h (Fig. S14), concomitant with a significant loss in the S. aureus viabilities. *, *P* < 0.05; **, *P* < 0.01. (*n *= 3).

Contact-mediated killing of other bacterial strains by S. marcescens’ is a hallmark of this strain, particularly due to its Type VI secretion system (T6SS) (22). Based on the draft genome sequence, S. marcescens RH10 also encodes many of the T6SS genes (Table S2). To explore if it is active in this new isolate, we conducted cell-to-cell contact experiments on agar plates with E. coli MG1655 as the recipient strain as describe previously (22), albeit on disks, using both S. marcescens RH10 and the nonpigmented S. marcescens Db10 (Table S1) (22, 23). These results are plotted in Fig. 2A. As reported previously, S. marcescens expresses its T6SS at 37°C but not at lower temperatures (22), explaining the much better killing efficiencies at the elevated temperature for both strains. Similar results were likewise obtained with S. marcescens RH10 Δ*pigA*, proving the activity of S. marcescens RH10 toward E. coli is not due to prodigiosin. Moreover, isogenic mutants in S. marcescens Db10, including TssE, ClpV (TssH) or Lip (TssJ), saw much of their activities toward E. coli MG1655 abolished, results that once more agree with the previous report (22). While the data show S. marcescens RH10 is capable of killing E. coli, ostensibly through its T6SS, no instances of T6SS-mediated killing of Gram-positive bacteria have been reported, presumably since their thick cell walls act as a barrier to this transport system (24). This was found to be the case as neither S. marcescens RH10 nor S. marcescens Db10 were biocidal toward S. aureus when cocultured at 37°C (Fig. 2B and Fig. S12, respectively).

Fig. 2B also shows that wild-type S. marcescens RH10 caused a 2550-fold (i.e., 99.96%) drop in the S. aureus viability when grown initially and then cocultured at 30°C, a loss that is much faster than that observed in the liquid culture assays (Fig. 1B). This faster killing is not really surprising, however, as the agar protocol employed is designed to increase cell-to-cell contact between the microbes. Once more, no killing of S. aureus occurred with the S. marcescens RH10 Δ*pigA* mutant, establishing further that prodigiosin is responsible for this activity. Consequently, we explored whether this was accomplished by the prodigiosin already present in S. marcescens RH10 or if *de novo* production of this secondary metabolite was necessary by UV-killing the S. marcescens RH10 cultures (Fig. S13) to prevent any additional synthesis of prodigiosin. As shown in Fig. 2C, UV-killing of this strain eliminated its bactericidal activities, indicating *de novo* synthesis is necessary for killing of S. aureus to be realized. While UV-killing of the S. marcescens RH10 Δ*pigA* mutant also led to a significantly better S. aureus growth, this is likely due to a reduced competition for nutrients rather than the production of some other inhibitory factor.

The necessity of *de novo* synthesis of prodigiosin was studied further in Fig. 2B and D, where the S. marcescens RH10 cultures were initially grown at either 30°C or 37°C and shifted to the other temperature for the contact killing assays. The rationale for doing this is S. marcescens is incapable of producing prodigiosin when grown at 37°C (25–27) (Fig. S14). As shown in Fig. 2B, when the culture temperature was shifted from 30°C to 37°C, killing of S. aureus by S. marcescens RH10 was significantly mitigated. Although a shift from 37°C to 30°C should allow this strain to generate prodigiosin, the S. aureus viabilities were only slightly lower and akin to those found when the same experiments were performed using nonpigmented strains (i.e., the Δ*pigA* mutant and S. marcescens Db10), suggesting the prodigiosin yields were still not sufficient for them to be bactericidal. Fig. 2D and S15 shows that this was the case, as longer times were needed when S. marcescens RH10 was initially grown at 37°C.

In conclusion, this study shows the prodigiosin-based antibacterial activities of S. marcescens are dependent on both cell-to-cell contact and *de novo* production of this secondary metabolite. Although we demonstrated prodigiosin can be released by S. marcescens RH10 within membrane vesicles budding off its cell surface, it is not biologically active in this form, nor was the S. marcescens RH10 spent media. These findings led us to conclude transfer must be contact-mediated, a fact we established using a protocol commonly employed to study T6SS activities. Within the genome of S. marcescens RH10 T6SS genes were identified, and experiments showed that this strain can effectively kill E. coli, proving this system is actively expressed. However, the T6SS had no apparent activity against S. aureus. Consequently, S. marcescens RH10 clearly possesses two different contact-based mechanisms, one engineered to kill Gram-positive strains (prodigiosin) under ambient temperatures, such as those on the skin of amphibians, and one for Gram-negative bacterial strains (T6SS), which is active at 37°C, making it the arsenal of choice for S. marcescens to combat bacterial competitors within the guts of warm-blooded animals.

### Data availability.

Raw filtered Flongle reads and the annotated assembly for *S. marcescens* RH10 isolate have been deposited under NCBI BioProject accession PRJNA741880 and Sequence Read Archive SRR14952065.
