# Supplementary material for: The Kiss of Death: Serratia marcescens Antibacterial Activities against Staphylococcus aureus Requires Both de novo Prodigiosin Synthesis and Direct Contact
Source: Microbiol Spectr. 2022 Apr 18;10(3):e00607-22. doi: 10.1128/spectrum.00607-22 (PMC9241871; doi:10.1128/spectrum.00607-22)
Supplement: SUPPLEMENTAL FILE 1 — Supplemental material. Download spectrum.00607-22-s001.pdf, PDF file, 0.6 MB [file spectrum.00607-22-s001.pdf]

Supplemental Information and Data

for

The kiss of death: *Serratia marcescens* antibacterial activities against *Staphylococcus aureus* requires both *de novo* prodigiosin synthesis and direct contact

Running title: Prodigiosin antibacterial activity is contact mediated

Sungbin Lim[1], Jihun Bhak[23], Sungwon Jeon[4], Wonsik Mun[1], Jong Bhak[2345], Seong Yeol Choi[1,]*, Robert J. Mitchell[1,]*

[1] Department of Biological Sciences, Ulsan National institute of Science and Technology (UNIST) Ulsan, 44919, South Korea

[2] Department of Biomedical Engineering, Ulsan National institute of Science and Technology (UNIST) Ulsan, 44919, South Korea

[3] Korean Genomics Center (KOGIC), Ulsan National Institute of Science and Technology (UNIST), Ulsan 44919, Republic of Korea

[4] Clinomics Inc., Ulsan 44919, Republic of Korea

[5] Personal Genomics Institute (PGI), Genome Research Foundation (GRF), Osong 28160, Republic of Korea.

Correspondence

Email – asterafe@gmail.com (CSY) & esgott@unist.ac.kr (RJM)

Materials and Methods

Bacterial Strains and Growth

All of the bacterial strains are listed in Table S1. *S. marcescens* RH10 was originally isolated from diluted aliquots of raw milk. After growth at 30ºC for 24 hours, a bright red colony that formed was isolated, grown and identified based on its 16S rDNA sequence (Fig S16). The type strain, *S. marcescens* ATCC 13880, was purchased from the Korean Collection for Type Cultures (KCTC) (Strain No. 42171) while *S. marcescens* Db10 and its isogenic mutants were kindly provided by Dr. Sarah Coulthurst from the University of Dundee. All of these strains were grown on lysogeny broth (LB) agar plates at 30ºC. For the experiments, each strain was grown in 20 ml of nutrient broth (NB) in 100 ml baffled flask at 30ºC, unless mentioned otherwise. Several other bacterial strains were also routinely grown, including *E. coli* MG1655 and a multidrug-resistant clinical isolate of *S. aureus* (1, 2), both of which were cultivated at 37ºC, with gentamicin (50 µg/ml) added to the *S. aureus* cultures.

Phylogenetic and Evolutionary Analysis by a Maximum Likelihood Method

The chromosomal DNA from *S. marcescens* RH10 was purified using the Genomic DNA Isolation, Flexible Kit (Nucleogen, South Korea) and the 16S gene was amplified with primers 27f and 1492r (Table S3). The 16S gene sequence was used to infer the evolutionary history of this isolate using the Maximum Likelihood method and Kimura 2-parameter model (3). The tree with the highest log likelihood (-5169.74) is shown. The percentage of trees in which the associated taxa clustered together is shown next to the branches. Initial tree(s) for the heuristic search were obtained automatically by applying Neighbor-Join and BioNJ algorithms to a matrix of pairwise distances estimated using the Maximum Composite Likelihood (MCL) approach, and then selecting the topology with superior log likelihood value. A discrete Gamma distribution was used to model evolutionary rate differences among sites (5 categories (+G, parameter = 0.2950)). The rate variation model allowed for some sites to be evolutionarily invariable ([+I], 38.67% sites). The tree is drawn to scale, with branch lengths measured in the number of substitutions per site. This analysis involved 23 nucleotide sequences, with a total of 1545 positions in the final dataset. Evolutionary analyses were conducted using MEGA X (4).

Purification of Prodigiosin

To quantify the amount of prodigiosin produced by the different *S. marcescens* strains, individual cultures were grown and at set times, samples were taken for optical density (600 nm) measurement, viability counts (using colony-forming units (CFU)) and to measure the amount of prodigiosin by HPLC. For the latter, the cells within the samples were pelleted by centrifugation (8,000 × g, 30 min at 4 °C), resuspended in an equal volume of acidified methanol (4% (v:v) 1M hydrochloric acid (HCl)) and shaken at 30°C for 24 hr. The solution was then transferred to fresh tube, separated by centrifugation (8,000 × g, 30 min at 4 °C). The supernatant was then vacuum filtered (0.22 µm; Millipore, USA) and concentrated in a rotary evaporator (N-1110, Eyela). The crude prodigiosin in the samples was dissolved in a water:ethyl acetate solution (1:1 (v:v)) and separated before the organic phase was concentrated once more in the rotary evaporator. The purified crude prodigiosin was finally dissolved in dimethyl sulfoxide and analyzed using high-performance liquid chromatography (HPLC 1200; Agilent, USA). For this, 10 µl samples of the ethanol extracted prodigiosin were separated on a C-18 column (Hypersil GOLD, 5 µm, 250 × 4.6 mm) at 30 ºC. The mobile phase used was 75% methanol (HPLC Grade, Sigma-Aldrich, USA) with 4% (v:v) 1M HCl added. The flow rate was 1 ml/min and detection was performed at 535 nm using an Agilent 1260 Infinity ELSD. The prodigiosin concentration within the extracts was determined using stocks of known concentration prepared with prodigiosin commercially available from Sigma-Aldrich (USA, Cat. No. P0103) as standards.

To perform NMR, the purified prodigiosin was solubilized in $CDCl_3$ and the spectra was measured using a Bruker 400 MHz FT-NMR AVANCE III HD. The NMR chemical shifts of prodigiosin were as follows: ^{1}H-NMR ($CDCl_3$, 400MHz, ppm) δ 0.83 (3H, t, H11″), 1.25 (2H, m, H9″), 1.28 (2H, m, H10″), 1.56 (2H, m, H8″), 2.41 (2H, t, H7″), 2.55 (3H, s, H6″), 4.01 (3H, s, OCH3), 6.09 (1H, d, H3′), 6.36 (1H, m, H3), 6.69 (1H, brd, H3″), 6.92 (1H, m, H4), 6.96 (1H, brs, H6′), 7.24 (1H, m, H2), 12.57 (1H, brs, H1), 12.74 (1H, brs, H1′); The signals from the protons in the molecule are noted and correspond to those published previously (5).

Co-Culture Experiments

The co-culture experiments were performed using either broth or agar systems. For the broth experiments, an overnight culture of either one of the *S. marcescens* strains or *E. coli* MG1655 was inoculated into 20 ml of sterile NB media in 100 ml baffled flask to an OD (600 nm) of 0.02. Into the same flask, an overnight culture of *S. aureus* was also introduced (10,000-fold dilution). The viabilities of the strains were measured at each time point by plating them on LB agar plates. Gentamicin was added to the agar plates as needed to specifically screen for *S. aureus*.

Direct contact experiments to study contact killing between *S. marcescens* and *S. aureus* were performed as described previously (6), with slight modification. Briefly, cultures of *S. marcescens*, *E. coli* and *S. aureus* were grown at the temperatures stated and their cells pelleted (13,000 x g, 1 min) and washed with dilute nutrient broth (0.1x NB) twice before being resuspended in fresh, sterile NB. The cultures were then mixed so the final *S. marcescens* or *E. coli* cell density was between 3~5x10^7 CFU/ml while that of *S. aureus* was 3~5x10^5 CFU/ml. From this, 50 µl was spotted onto a sterile 0.22µm filter disk on an LB agar plate (Figs S14 and S15). The direct contact experiments were typically performed for 6 hr at 30ºC, after which the filter with the bacteria attached was aseptically removed, the bacteria dispersed into fresh NB media and the viabilities of the two species determined by plate counts. Parallel experiments to evaluate the viabilities at other time points were also performed.

Flongle Library Construction and Whole-Genome Sequencing

Genomic DNA was extracted from an overnight culture of *S. marcescens* RH10 using the Genomic DNA Isolation, Flexible Kit (Nucleogen, South Korea), according to the manufacture's recommended protocol. Whole-genome sequencing was performed using the Oxford Nanopore Technologies (ONT) Flongle FLO-FLG001 (R9.4.1) flow cell and Ligation sequencing kit (SQK-LSK109) as described previously (7).

Base Calling and Read Preparation

Flongle raw signals were base called using Guppy (v4.0.11) with the high-accuracy model. The base-called reads with low-quality score (< Phred quality score 7) were filtered simultaneously. Adapter

sequences were trimmed using porechop (v0.2.4, https://github.com/rrwick/Porechop). Length statistics of Flongle reads was calculated using NanoPlot (v1.33.0) (8).

De Novo Assembly and Genome Annotation

We generated three different primary *de novo* assemblies from the ONT's Flongle reads using Flye (v2.8.1) (9, 10), Raven (v1.2.2) (11), and wtdbg2 (-x ont -g 5.1m, not polished) (v2.5) (12). Each assembly was clustered with the other two assemblies and reconciled into a circular form to construct a bacterial genome. A consensus assembly was generated from the multiple sequence alignment of the three assemblies and read alignments by selecting variants with high read alignment scores and low hamming distance using Trycycler (v0.3.3, https://github.com/rrwick/Trycycler/tree/v0.3.3) (13). To improve the base accuracy of the consensus assembly, we polished the consensus assembly using medaka (v1.1.1). Contiguity statistics of the final assembly were assessed by QUAST (v5.0.2) (14, 15). BUSCO (v4.1.2) (16) with the enterobacterales odb10 database was used to assess the gene completeness in the assembly. Genome annotation was also performed by PGAP (17-19).

We obtained 99,246 Flongle base-called reads with an average length of 4,695 bp with a read N50 (the read length such that reads of this length or greater sum to at least half the total bases) of 16,420 bp and a total sequencing output of 465 Mb (Table S4). After adapter trimming, the preprocessed reads were assembled according to the methodology described above and submitted to the National Center for Biotechnology Information (NCBI) sequence read archives (SRA) (Accession Numbers SRR14952065, PRJNA741880). The assembled genome of *S. marcescens* strain RH10 contained one contig in a circular form that was 5.13 Mb in size with a G + C content of 59.53% (Fig S6), which is similar to the Type strain, *S. marcescens* ATCC 13880 (Genbank JOVM01000004.1). The genome completeness assessment by BUSCO using the 440 conserved Enterobacterales single-copy orthologs showed that 423 (96.2%) genes were completely present, seven (1.6%) genes were fragmented and ten (2.3%) genes were missing in our assembly (Fig S7). We predicted 4,907 genes are present in *S. marcescens* RH10, including 4,480 protein coding genes, 22 complete rRNA subunits (8 - 5S, 7 - 16S, and 7 - 23S), 91 tRNAs, 15 noncoding RNAs and 299 pseudogenes.

Construction of a *S. marcescens* RH10 *pigA* Knock-Out Mutant

To construct a Δ*pigA* mutant within *S. marcescens* RH10, the suicidal vector pKNOCK-Km (20) was used. An internal segment of the *pigA* gene was first amplified by polymerase chain reaction (PCR) using primers *pigA* F, *pigA* R1 and *pigA* R2 (Table S3) and cloned into pKNOCK-Km using the EMP cloning technique (21). The resulting plasmid, *i.e.*, pKNOCK-*pigA*, was then transformed via electroporation into electrocompetent *S. marcescens* RH10. After 24 hr at 30ºC, white colonies grew on LB agar plates containing 50 µg/ml kanamycin. Confirmation was performed by PCR (Fig S8) using the KanR, *pigA*, and *pigD* conf primer pairs (Table S3) and 16S rDNA sequencing analyses. The DNA ladder used was the 1 kb (+) ladder (Enzynomics, Korea).

Purification of Membrane Vesicles from *S. marcescens* RH10

To purify membrane vesicles (MVs), 300 ml bacterial cultures were grown in NB medium at 30°C and 250 rpm in 1 L flasks for 24 h. The bacterial cells and debris were removed by low-speed centrifugation (8,000 × g, 30 min) and the medium was sterilized by passing it through a 0.22 µm filter (Millipore, USA). The cell-free medium was concentrated to ~5 ml using a 100-kDa MWCO Amicon filter (USA). The concentrated MVs were pelleted by ultracentrifugation (14,0000 × g, 3 h, 4 °C) and suspended in 25 mM HEPES (pH 7.5). They were then mixed with OptiPrep (Sigma Aldrich, USA) to generate a 40% (vol/vol) OptiPrep solution in a total volume of 2 ml. The samples were loaded into a 13.2-ml ultracentrifuge tube, and lower-concentration OptiPrep solutions were layered on top [2 ml (40%), 2 ml (30%), 2 ml (25%), 2 ml (20%), 1 ml (15%) and finally 0.5 ml (0%)]. The samples were centrifuged (100,000 × g, 16 h, 4°C) in a swinging-bucket rotor, after which a 2 ml fraction containing the red band was collected, diluted into 25 mM HEPES ((4-(2-hydroxyethyl)-1-piperazineethanesulfonic acid, pH 7.5) and recovered the MVs via ultracentrifugation (14,0000 × g, 3 h, 4°C). The recovered pellets were then re-suspended in 25 mM HEPES (pH 7.5) and the amount of prodigiosin present within them was determined using acidified methanol as described above.

TEM Analyses

Imaging of the cells and purified MVs was performed as described previously (1, 22, 23). Briefly, 2 µl of the bacterial culture or purified MVs were placed on carbon-coated grids (Polysciences, Inc., USA). The samples were treated with 2.5% glutaraldehyde for 1 h, washed in 25 mM HEPES (pH 7.5) and then stained with a 4% osmium tetroxide solution (Sigma-Aldrich, USA) for 1 h. The samples were sequentially washed with HEPES (pH 7.5) containing increasing amounts of ethanol (50%, 60%, 80%, 90%, 95% and 100%). After the final wash, they were examined under a transmission electron microscope (JEOL 1200EX, USA) using 10,000 x magnification.

Activity of *S. marcescens* Spent Media and Membrane Vesicle Towards *S. aureus*

For these tests with the spent media, overnight cultures of the different *S. marcescens* strains and *E. coli* MG1655 were sterile-filtered (0.22 µm) and mixed with NB media 1:1 (v:v). To this, overnight cultures of *S. aureus* were diluted to an initial cell density of 3~5x10^5 CFU/ml and cultured for 24 hr at 30ºC and 250 rpm, after which their viabilities (CFU/ml) were measured.

The antibacterial activities of the purified MVs *S. marcescens* RH10 were evaluated and compared against that of purified crude prodigiosin, each tested using the same concentrations. These tests and analyses were performed as described previously (1). After 6 h at 30ºC, the surviving *S. aureus* populations in each of the samples were enumerated by serially dilution and growth on LB agar plates overnight at 37ºC.

UV Treatment

To kill the *S. marcescens* cultures, they were exposed to UV light within a biosafety cabinet. For this, 5 ml of overnight cultures, grown as described above in NB media, were pelleted by centrifugation (7,200 × g, 5 min), washed twice with and adjusted to OD 1.0 in DNB media. From this, 5ml were transferred into sterile a petridish and exposed to UV for 60 minutes, leading to a significant loss (nearly 4-log) in their viabilities (Fig S13). These cultures were then used in the co-culture assays as described above.

Statistical Analyses

All tests were conducted in triplicate and the standard deviations among the samples are indicated with error bars on the graphs. Statistical analysis was done using the Student's t-test to compare two sets of results and statistically different results are indicated within the graphs (*, ** or "b", *** for *p*-values of less than 0.05, 0.01 and 0.001 respectively).

Table S1. Bacterial strains used in this study

**Bacterial Strain**	**Description**	**References**
Staphylococcus aureus	Multidrug resistant clinical isolate	(1)
Serratia marcescens RH10	Newly isolated, strong producer of prodigiosin	This study
Serratia marcescens RH10 Δ*pigA*	Isogenic, pigmentless variant of *S. marcescens* RH10	This study
Serratia marcescens ATCC 13880	Type-strain; Weak prodigiosin producer	
Serratia marcescens Db10	Pigmentless strain of *S. marcescens*	(6, 24)
Serratia marcescens Db10 Δ*lip*	T6SS-deficient strain of *S. marcescens* Db10	(6)
Serratia marcescens Db10 Δ*clpV*	T6SS-deficient strain of *S. marcescens* Db10	(6)
Serratia marcescens Db10 Δ*tssE*	T6SS-deficient strain of *S. marcescens* Db10	(6)
Escherichia coli MG1655	Non-pathogenic Gram-negative bacterial strain	

Table S2. Main operon protein sequences (black) are highly homologous with other proteins found in *Serratia*. Orphan (independent) genes (listed in blue) are also highly homologous, except one TssI gene.

Protein	Description	% Identity [a]
TssI	Type VI secretion system tip protein VgrG	99 (777/779)
TssJ	Type VI secretion system lipoprotein	100
TssK	Type VI secretion system baseplate subunit	100
DotU (TssL?)	DotU family type VI secretion system protein	100
TssM	Type VI secretion system membrane subunit	99 (1207/1211)
TagF	Type VI secretion system-associated protein	99 (238/239)
TssA	Type VI secretion system protein	99 (340/343)
TssB	Type VI secretion system contractile sheath small subunit	100
TssC	Type VI secretion system contractile sheath large subunit	100
Hcp	Type VI secretion system tube protein	100
Tae4	Type VI secretion system amidase effector protein	100
Tai4	Type VI secretion system amidase immunity protein	100
tmp_003025	Type VI secretion protein	100
Hypothetical	Hypothetical protein	100
TagH	Type VI secretion system-associated FHA domain protein	Frameshift
Phosphatase	Serine/threonine-protein phosphatase	100
tmp_003029	Type VI secretion system-associated protein	100
tmp_003030	Protein of avirulence locus ImpE	99 (264/267)
tmp_003031	GPW/gp25 family protein	100
TssF	Type VI secretion system baseplate subunit	100
TssG	Type VI secretion system baseplate subunit	99 (355/356)
TssH	Type VI secretion system ATPase	99 (881/884)
Kinase	Serine/threonine protein kinase	99 (480/481)
TssI	Type VI secretion system tip protein VgrG	99 (634/641)
TssI	Type VI secretion system tip protein VgrG	79 (495/625)
Hcp	Type VI secretion system tube protein	100
Hcp	Type VI secretion system tube protein	99 (159/160)

[a] – Based on the amino acid sequences from homologous proteins found in other *S. marcescens* strains listed within the National Center for Biotechnology Center (NCBI) website. The numbers in parenthesis indicate the number of amino acids within the coding region, *i.e.*, annotated gene, that are identical out of the total.

Table S3. Primer used in both the construction of the pKNOCK-*pigA* plasmid and to confirm the *S. marcescens* RH10 Δ*pigA* mutant, as shown in Fig S3. The expected sizes are listed for the latter.

Primer	Sequence	Description
27f	AGAGTTTGATCMTGGCTCAG	16S rDNA gene sequencing
1492r	GGTTACCTTGTTACGACTT	16S rDNA gene sequencing
pigA F	GCACCAGACCGAGTTCTTGA	pKNOCK-*pigA* recombination
pigA R1	TTAATTCGACGCGTCCTCGGGGCATGAATGCGCTCGATAC	pKNOCK-*pigA* recombination
pigA R2	GATCCACTAGTTCTAGAGCGGC	pKNOCK-*pigA* recombination
KanR confF	GATGGATTGCACGCAGGTTC	Confirmation primers
KanR confR	TAAAGCACGAGGAAGCGGTC	for the kanamycin gene **(718 bp)**
pigA confF	AGCAGGCTCTAAGCGAATCC	Confirmation primers
pigA confR	CTTGCGGGCGACAATTTCTT	for the *pigA* gene **(1108 bp)**
pigD confF	GCCCAGTATAGCCGGACATC	Confirmation primers
pigD confR	GACTGTTCCCCCGCCATAAA	for the *pigD* gene **(1741 bp)**

Table S4. Overview of Flongle raw read statistics of *S. marcescens* strain RH10

Total number of reads	99,246
Total bases (bp)	465,982,942
Read length N50 (bp)	16,420
Mean read length (bp)	4,695.20
Mean read quality	11.1
Median read length (bp)	1,070.00
Median read quality	11.2

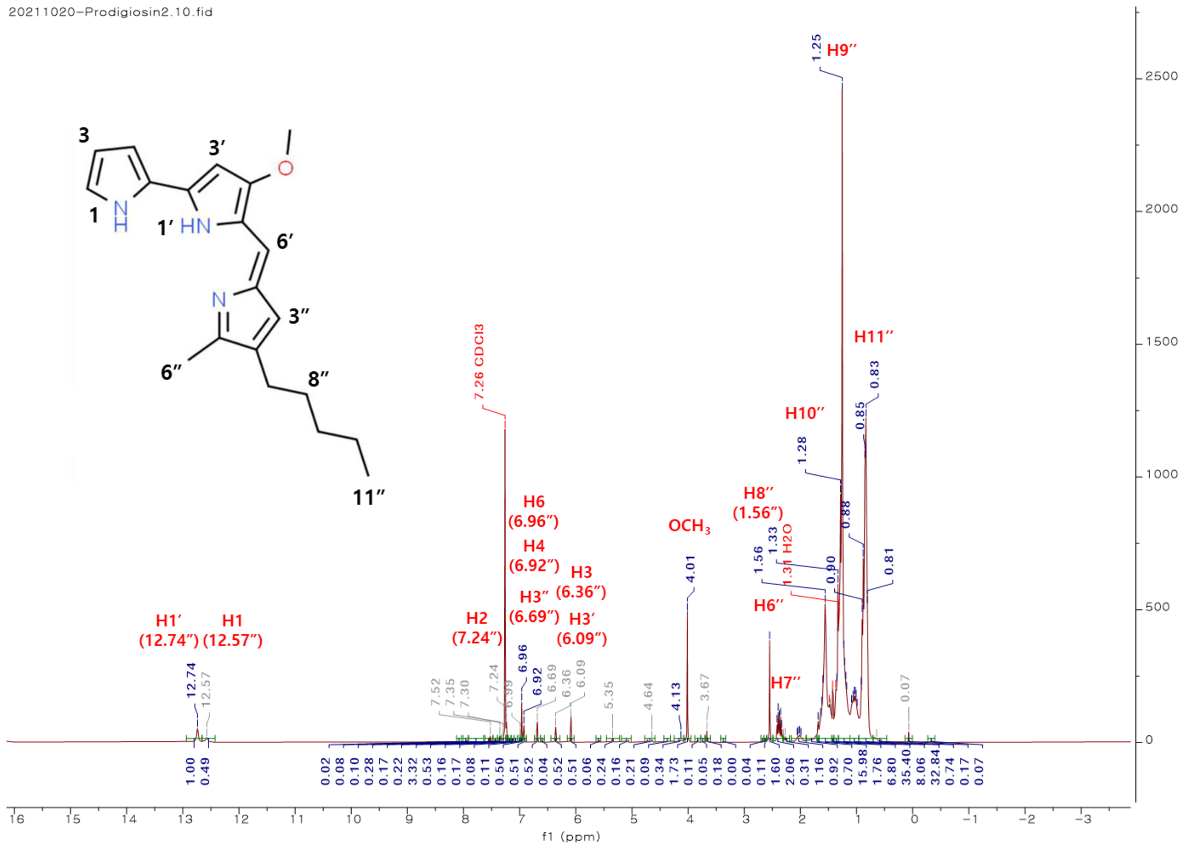

Fig S1. *S. marcescens* RH10 synthesizes prodigiosin. This image is the ^{1}H-NMR spectra of the purified prodigiosin in $CDCl_3$. The signals from the protons and carbons in the molecule are noted and correspond to those published previously (5).

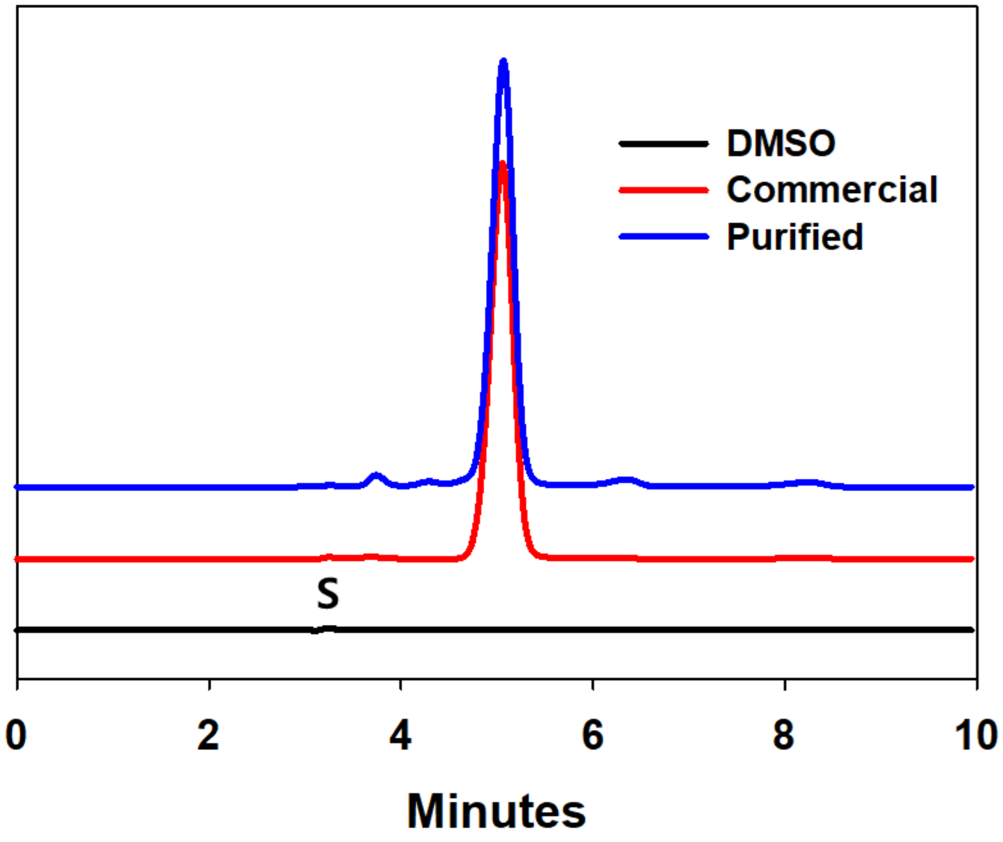

Fig S2. HPLC analysis of the prodigiosin extracted from cultures of *S. marcescens* RH10, showing the presence of prodigiosin (maximum peak at 5.07 min). The solvent front (Peak S) is barely visible at 3.2 min. A plot generated using commercially available prodigiosin (Sigma-Aldrich (Cat. # P0103)) extracted from *Serratia marcescens* is also provided for comparison, with a maximum peak at 5.05 min. The concentration of prodigiosin in each sample was 25 µg/ml.

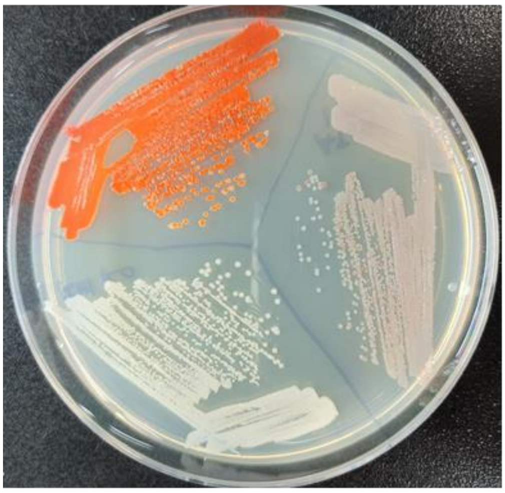

Fig S3. Colony morphologies of the different *S. marcescens* cultures, showing the varying prodigiosin production levels for each. This plate was grown at 30ºC for 48 h.

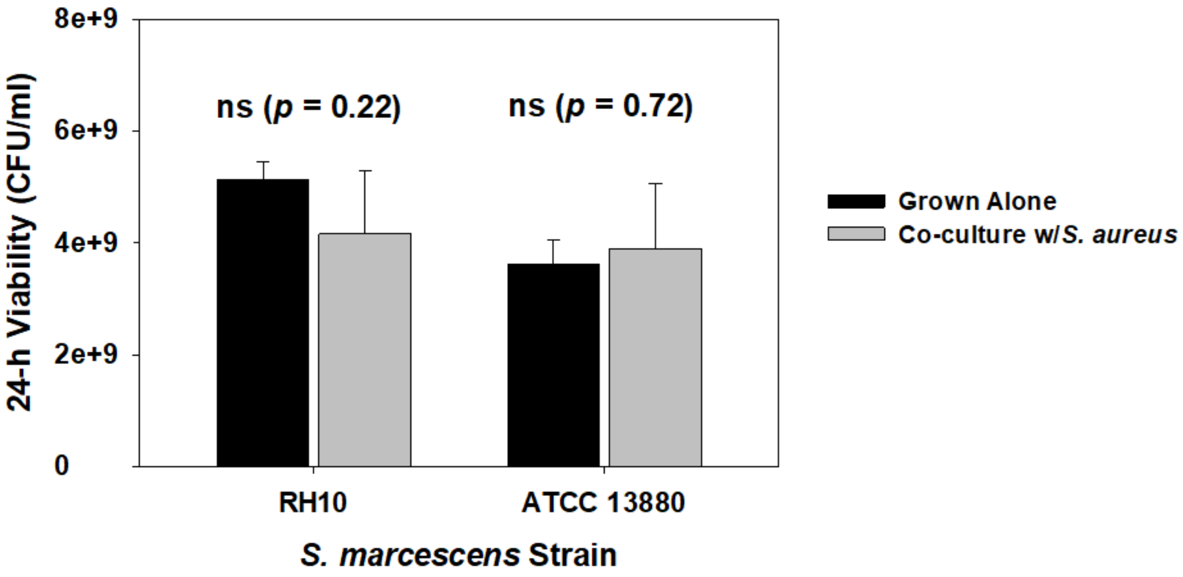

Fig S4. 24-h *S. marcescens* viabilities are not significantly different when grown alone or in co-cultures with *S. aureus*. (n = 3)

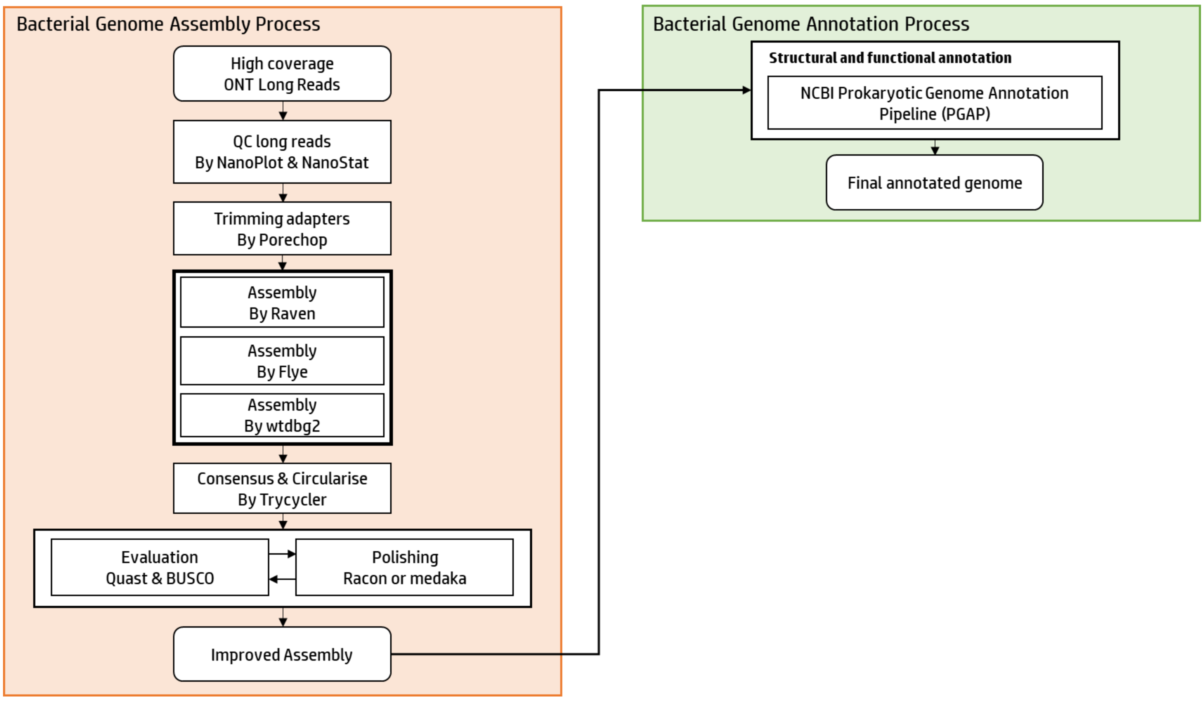

Fig S5. Bacterial Genome Assembly and Annotation Flowchart. This is the flowchart used to assemble, annotate and analyze the genome sequence for *S. marcescens* RH10 generated using the Oxford Nanopore Technologies flongle-based system.

	Flye	Raven	wtdbg2	Trycycler	Trycycler + medaka
**Number of contig**	1	1	1	1	1
**Total bases in assemblies (bp)**	5,132,487	5,133,978	5,126,780	5,136,047	5,136,041
**Maximum contig length (bp)**	5,132,487	5,133,978	5,126,780	5,136,047	5,136,041
**N50 (bp)**	5,132,487	5,133,978	5,126,780	5,136,047	5,136,041
**L50 (bp)**	1	1	1	1	1
**Number of N's per 100kbp**	0	0	0	0	0
**G + C (%)**	59.53	59.53	59.53	59.53	59.53

Fig S6. The *S. marcescens* RH10 whole genome assembly shows good contiguity. The results indicate on one contig per replicon and the length (5,136,041 bp) of the final assembly draft genome (Trycycler+medaka) is quite similar with that of *S. marcescens* ATCC 13880 (5,131,648 bp).

	Flye	Raven	wtdbg2	Trycycler	Trycycler + medaka
**Complete BUSCOs**	373 (84.8%)	378 (85.9%)	249 (56.6%)	378 (85.9%)	423 (96.1%)
**Complete and single-copy BUSCOs**	373 (84.8%)	378 (85.9%)	249 (56.6%)	378 (85.9%)	423 (96.1%)
**Complete and duplicated BUSCOs**	0 (0.0%)	0 (0.0%)	0 (0.0%)	0 (0.0%)	0 (0.0%)
**Fragmented BUSCOs**	43 (9.8%)	39 (8.9%)	118 (26.8%)	41 (9.3%)	7 (1.6%)
**Missing BUSCOs**	24 (5.4%)	23 (5.2%)	73 (16.6%)	21 (4.8%)	10 (2.3%)
**Total BUSCO groups searched**	440	440	440	440	440

Fig S7. Benchmarking Universal Single-Copy Ortholog (BUSCO) scores for the *S. marcescens* RH10 genome based on the analyses performed, showing high complete BUSCOs (96.1%) and low fragmented BUSCOs (1.6%) for the final assembly draft genome (Trycycler+medaka).

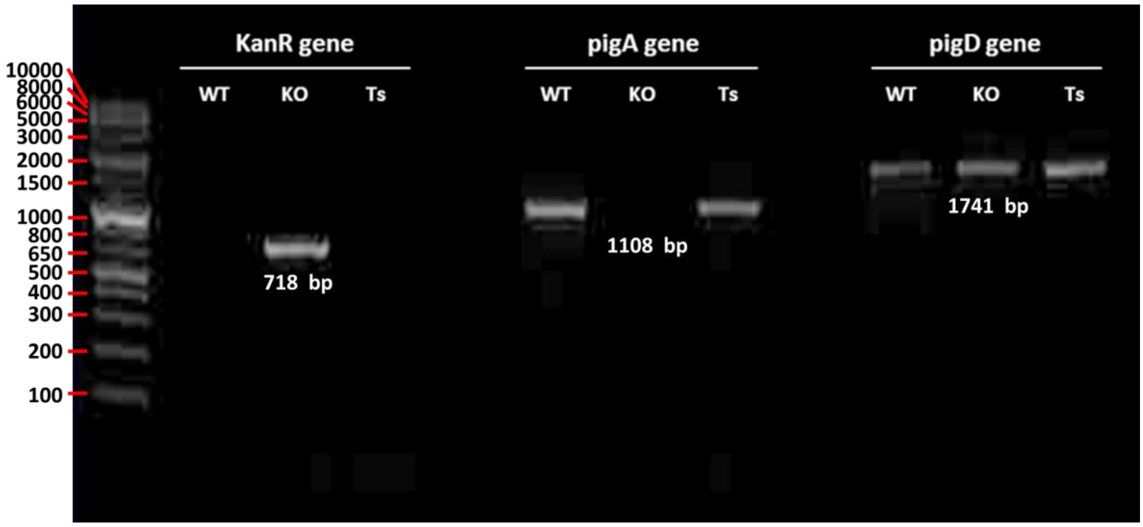

Fig S8. Confirmation that the *pigA* gene was knocked-out in *S. marcescens* RH10 Δ*pigA* (KO). PCR was performed using genomic DNA isolated from the mutant strain as well as wild-type *S. marcescens* RH10 (WT) and the type strain *S. marcescens* ATCC 13880 (Ts). These results verify the prodigiosin-less phenotype of *S. marcescens* RH10 Δ*pigA* seen in Fig S1. Marker – 1 kb (+) ladder (Enzynomics, Korea)

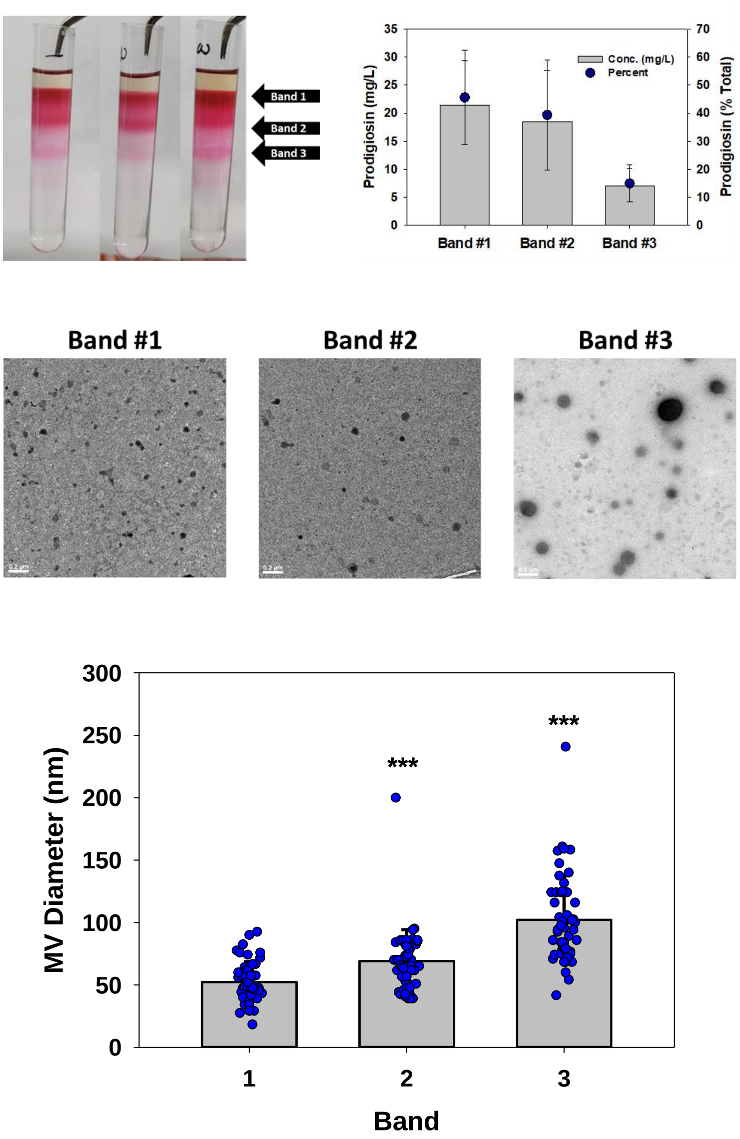

Fig S9. Purification and characterization of the MVs from *S. marcescens* RH10.

(**Upper left**) Images of the Optiprep gradient after ultracentrifugation, showing the presence of three distinct bands in the independent samples.

(**Upper right**) The contents of each were purified and the prodigiosin present quantified, showing Band #1 and Band #2 had had similar concentrations, which agrees with the pictures of the tubes, while Band #3 had much less (approximately 2.5- to 3-fold) (n = 3).

(**TEM images**) Representative TEM images of the MVs present in each band, showing the presence of much larger particles in Band #3. The size bars in each image are 0.2 µm.

(**Bottom**) The average MVs diameters for each are provided. (n = 50)

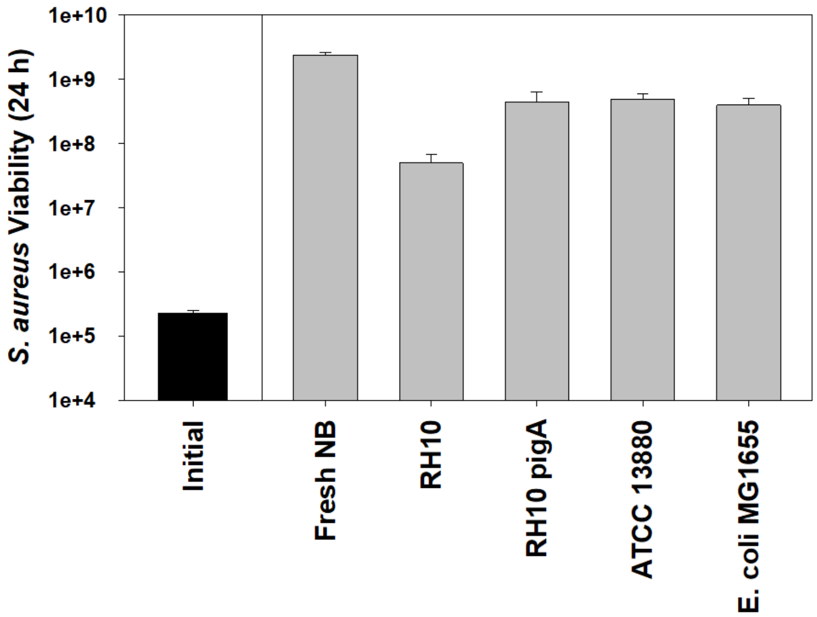

Fig S10. Spent media effects on the 24-h *S. aureus* population densities. The results show none of the *S. marcescens* spent media was bactericidal towards the *S. aureus* strain, even that from *S. marcescens* RH10. In each case, the spent media was filter-sterilized (0.22 µm). (*n* = 3)

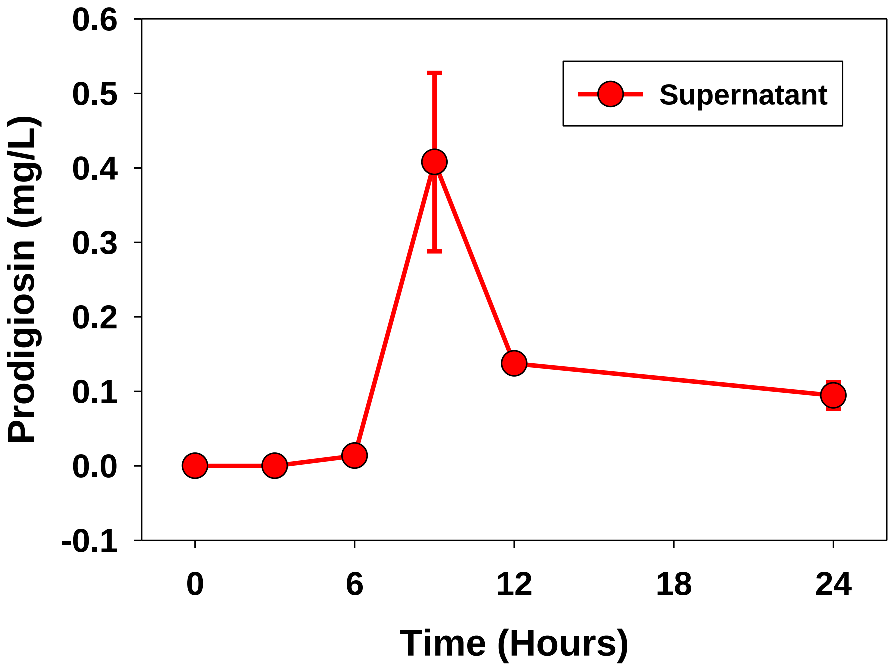

Fig S11. Prodigiosin present in the supernatant as *S. marcescens* RH10 grows. The results show a spike around nine hours. Based on the concentration effects in Fig. 1E, this concentration may have a mild effect, potentially leading to the reduced growth shown in Fig. S10 without being overtly bactericidal.

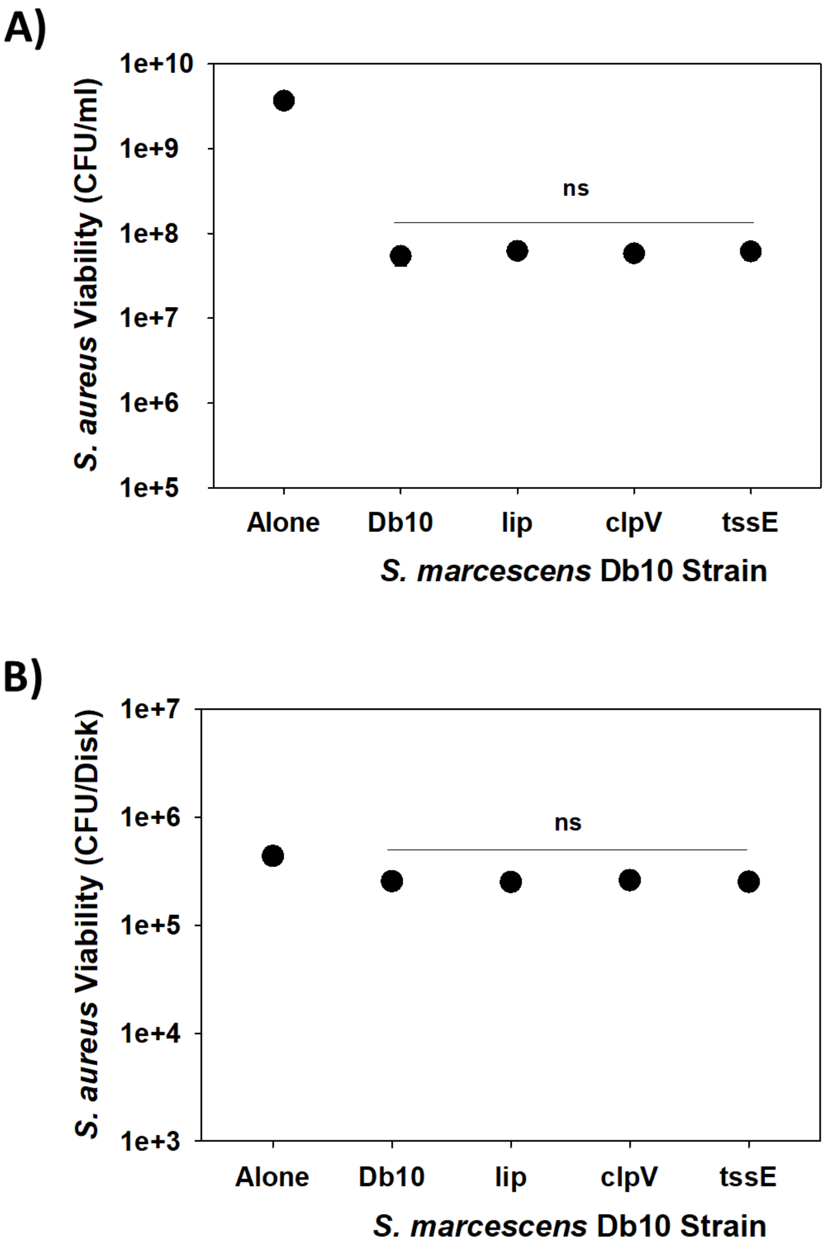

Fig S12. *S. marcescens* Db10 type VI secretion system is not active against *S. aureus*. Contact-killing assays were performed on the filter disks at either (A) 37ºC or (B) 30ºC, with neither showing any indication of bactericidal activities with either the wild-type *S. marcescens* Db10 or any of its isogenic mutants. ns – not significantly different.

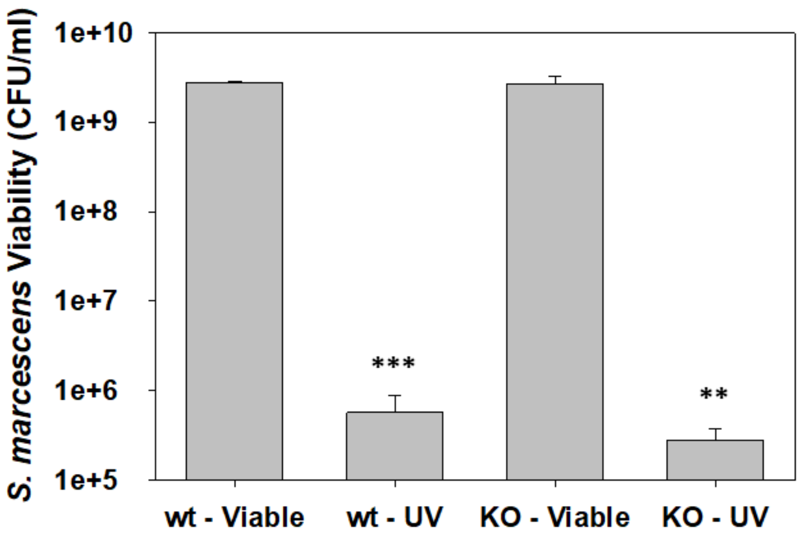

Fig S13. UV-killing of the wild-type and Δ*pigA* *S. marcescens* RH10 cultures. ($n = 3$) ** - $p < 0.01$; *** - $p < 0.001$

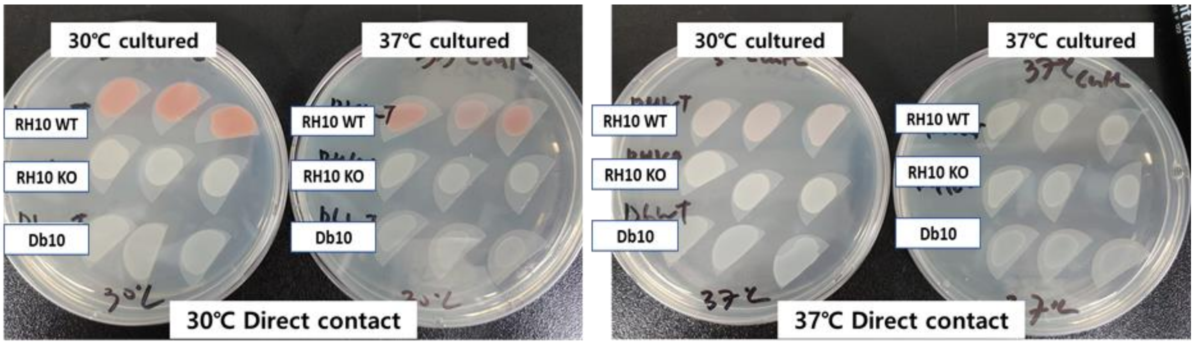

Fig S14. Filter-based direct contact experiments. The *S. marcescens* strains were initially grown at the temperatures indicated at the top of the pictures before being diluted and mixed with the *S. aureus* cultures and spotted onto the filter disks as shown. The mixed cultures on the filters were then grown at the temperature indicated on the bottom of the images. These images were captured after a six-hour incubation, showing the clear production of prodigiosin by wild-type *S. marcescens* RH10 (RH10 WT), and only at 30ºC, as opposed to the *S. marcescens* RH10 Δ*pigA* mutant (RH10 KO) or *S. marcescens* Db10 (Db10), a pigmentless strain, which remained colorless in all cases. (*n* = 3)

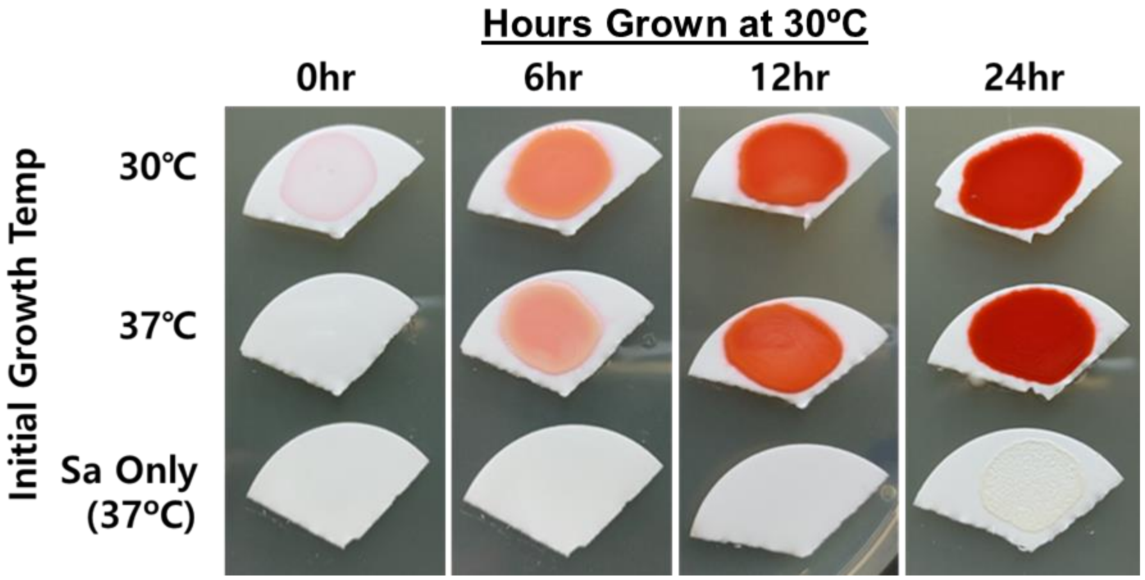

Fig S15. *De novo* prodigiosin from *S. marcescens* RH10 cultures grown initially overnight at either 30 or 37ºC (*y*-axis). All of these cultures were grown at 30ºC in these tests. This figure shows representative images of the filter disks for each culture, showing the color change development in the direct contact cultures as they were incubated at 30ºC. Note the initial orange hue with the 30ºC-cultivated *S. marcescens* RH10 culture while the 37º-cultivated culture was initially pigmentless (0 hours), helping to explain the more rapid killing of *S. aureus* with the former in Fig 2d. Images were taken at the same times as and just prior to performing the viability checks in Fig 2D. (n = 3)

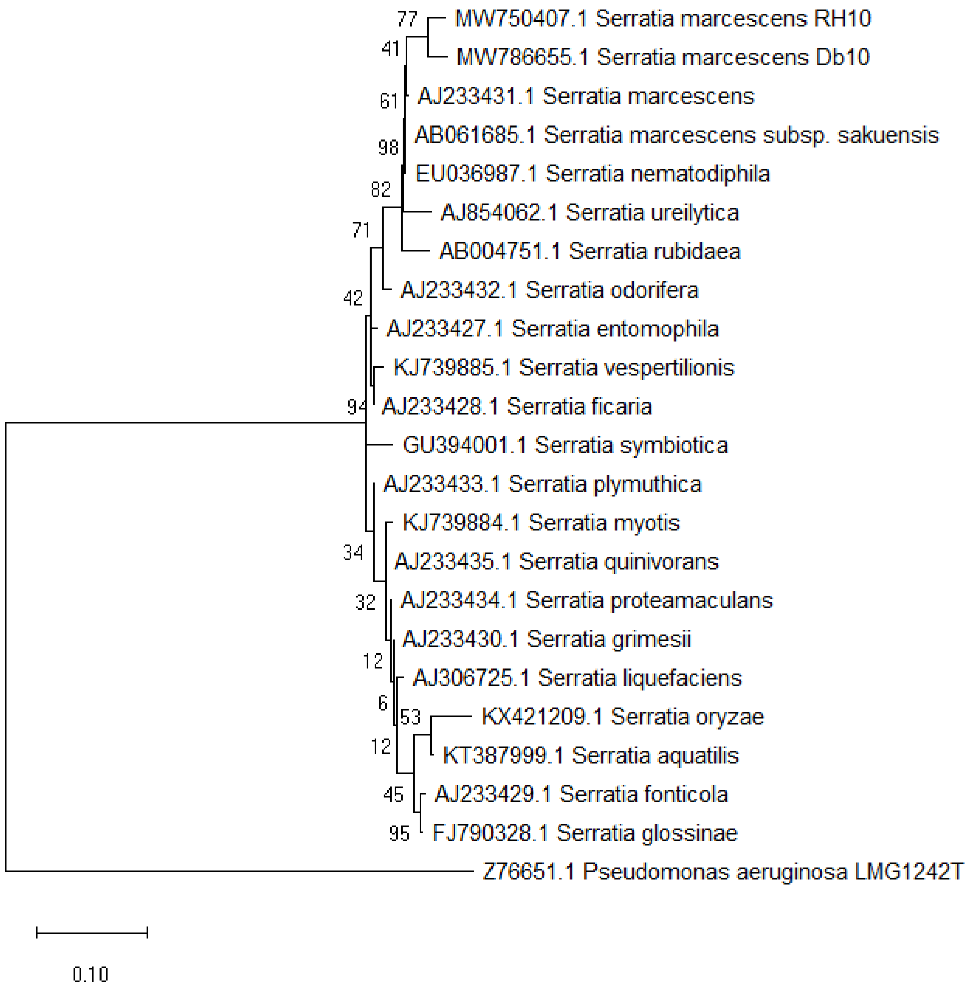

Fig S16. Phylogenetic tree of the newly isolated *S. marcescens* RH10, showing its relationship with *S. marcescens* ATCC 13880 and *S. marcescens* Db10. Evolutionary analysis by Maximum Likelihood method

References

1. Choi SY, Lim S, Cho G, Kwon J, Mun W, Im H, Mitchell RJ. 2020. Chromobacterium violaceum delivers violacein, a hydrophobic antibiotic, to other microbes in membrane vesicles. Environmental Microbiology 22:705-713.
2. Choi SY, Kim S, Lyuck S, Kim SB, Mitchell RJ. 2015. High-level production of violacein by the newly isolated Duganella violaceinigra str. NI28 and its impact on Staphylococcus aureus. Scientific Reports 5.
3. Kimura M. 1980. A simple method for estimating evolutionary rates of base substitutions through comparative studies of nucleotide sequences. Journal of Molecular Evolution 16:111-120.
4. Kumar S, Stecher G, Li M, Knyaz C, Tamura K, Battistuzzi FU. 2018. MEGA X: Molecular Evolutionary Genetics Analysis across Computing Platforms. Molecular Biology and Evolution 35:1547-1549.
5. Song M-J, Bae J, Lee D-S, Kim C-H, Kim J-S, Kim S-W, Hong S-I. 2006. Purification and characterization of prodigiosin produced by integrated bioreactor from Serratia sp. KH-95. Journal of Bioscience and Bioengineering 101:157-161.
6. Murdoch SL, Trunk K, English G, Fritsch MJ, Pourkarimi E, Coulthurst SJ. 2011. The Opportunistic Pathogen Serratia marcescens Utilizes Type VI Secretion To Target Bacterial Competitors. Journal of Bacteriology 193:6057-6069.
7. Kwak W, Han Y-H, Seol D, Kim H, Ahn H, Jeong M, Kang J, Kim H, Kim TH. 2020. Complete Genome of Lactobacillus iners KY Using Flongle Provides Insight Into the Genetic Background of Optimal Adaption to Vaginal Econiche. Frontiers in Microbiology 11.
8. De Coster W, D'Hert S, Schultz DT, Cruts M, Van Broeckhoven C. 2018. NanoPack: visualizing and processing long-read sequencing data. Bioinformatics 34:2666-2669.
9. Lin Y, Yuan J, Kolmogorov M, Shen MW, Chaisson M, Pevzner PA. 2016. Assembly of long error-prone reads using de Bruijn graphs. Proceedings of the National Academy of Sciences 113:E8396-E8405.
10. Kolmogorov M, Yuan J, Lin Y, Pevzner PA. 2019. Assembly of long, error-prone reads using repeat graphs. Nature Biotechnology 37:540-546.
11. Vaser R, Šikić M. 2021. Raven: a de novo genome assembler for long reads. bioRxiv doi:10.1101/2020.08.07.242461.
12. Ruan J, Li H. 2019. Fast and accurate long-read assembly with wtdbg2. Nature Methods 17:155-158.
13. Wick RR, Judd LM, Cerdeira LT, Hawkey J, Méric G, Vezina B, Wyres KL, Holt KE. 2021. Trycycler: consensus long-read assemblies for bacterial genomes. Genome Biology 22:266.
14. Gurevich A, Saveliev V, Vyahhi N, Tesler G. 2013. QUAST: quality assessment tool for genome assemblies. Bioinformatics 29:1072-1075.
15. Mikheenko A, Valin G, Prjibelski A, Saveliev V, Gurevich A. 2016. Icarus: visualizer for de novo assembly evaluation. Bioinformatics 32:3321-3323.
16. Seppey M, Manni M, Zdobnov EM. 2019. BUSCO: Assessing Genome Assembly and Annotation Completeness, p 227-245, Gene Prediction doi:10.1007/978-1-4939-9173-0_14.
17. Tatusova T, DiCuccio M, Badretdin A, Chetvernin V, Nawrocki EP, Zaslavsky L, Lomsadze A, Pruitt KD, Borodovsky M, Ostell J. 2016. NCBI prokaryotic genome annotation pipeline. Nucleic Acids Research 44:6614-6624.
18. Haft DH, DiCuccio M, Badretdin A, Brover V, Chetvernin V, O'Neill K, Li W, Chitsaz F, Derbyshire MK, Gonzales NR, Gwadz M, Lu F, Marchler GH, Song JS, Thanki N, Yamashita RA, Zheng C, Thibaud-Nissen F, Geer LY, Marchler-Bauer A, Pruitt KD. 2018. RefSeq: an update on prokaryotic genome annotation and curation. Nucleic Acids Research 46:D851-D860.
19. Li W, O'Neill KR, Haft DH, DiCuccio M, Chetvernin V, Badretdin A, Coulouris G, Chitsaz F, Derbyshire Myra K, Durkin AS, Gonzales NR, Gwadz M, Lanczycki Christopher J, Song JS, Thanki

N, Wang J, Yamashita Roxanne A, Yang M, Zheng C, Marchler-Bauer A, Thibaud-Nissen F. 2021. RefSeq: expanding the Prokaryotic Genome Annotation Pipeline reach with protein family model curation. Nucleic Acids Research 49:D1020-D1028.

20. Alexeyev MF. 1999. The pKNOCK Series of Broad-Host-Range Mobilizable Suicide Vectors for Gene Knockout and Targeted DNA Insertion into the Chromosome of Gram-Negative Bacteria. BioTechniques 26:824-828.

21. Isalan M, Ulrich A, Andersen KR, Schwartz TU. 2012. Exponential Megapriming PCR (EMP) Cloning—Seamless DNA Insertion into Any Target Plasmid without Sequence Constraints. PLoS ONE 7.

22. Im H, Lee S, Soper SA, Mitchell RJ. 2017. Staphylococcus aureusextracellular vesicles (EVs): surface-binding antagonists of biofilm formation. Molecular BioSystems 13:2704-2714.

23. Monnappa AK, Bari W, Seo JK, Mitchell RJ. 2018. The Cytotoxic Necrotizing Factor of Yersinia pseudotuberculosis (CNFy) is Carried on Extracellular Membrane Vesicles to Host Cells. Scientific Reports 8.

24. Cornelis P, Gerc AJ, Song L, Challis GL, Stanley-Wall NR, Coulthurst SJ. 2012. The Insect Pathogen Serratia marcescens Db10 Uses a Hybrid Non-Ribosomal Peptide Synthetase-Polyketide Synthase to Produce the Antibiotic Althiomycin. PLoS ONE 7.
